# Optimization of induction and hairy root culture establishment in two mullein species, *Verbascum erianthum* and *Verbascum stachydiforme*

**DOI:** 10.1038/s41598-024-56331-8

**Published:** 2024-03-07

**Authors:** Soniya Amini, Mohammad Fattahi, Hossein Nazemiyeh

**Affiliations:** 1 Department of Horticulture, Faculty of Agriculture, Urmia University, Urmia, Iran; 2 Research Center for Pharmaceutical Nanotechnology, Faculty of Pharmacy, Tabriz University of Medical Sciences, Tabriz, Iran

**Keywords:** *Agrobactrium rhizogenes*, Transformed root, *Verbascum*, Bacterial strain, Browning, Plant biotechnology, Biochemistry

## Abstract

The genus *Verbascum*, belonging to the family Scrophulariaceae, has a significant center of diversity in Iran. Two of its species, *V. erianthum and V. stachydiforme*, originate in the Iranian-Turanian region, but no studies have been conducted on the induction of their hairy roots. This genus is a valuable source of biologically active compounds such as iridoid glycosides and flavonoids. Hairy root culture is a suitable technique for the production and accumulation of secondary metabolites. Three different studies were conducted to optimize the induction and establishment of hairy roots. In the first experiment, the influence of explant type (leaf and hypocotyl), six infection methods, and co-culture time (48 and 72 h) on the efficiency of hairy root induction was investigated. The results showed that the highest hairy root induction (68.18%) was observed in the leaf explants inoculated by direct infection with three wounds within 72 h co-culture time. In the second experiment, the effect of four *Agrobacterium rhizogenes* strains (ATCC 15834, A4, A7, and A13) and leaf age (14, 21, and 28 days) on transformation efficiency and some morphological traits examined in both species were studied. The high transformation efficiency of hairy root (80.55%) was detected in the 21-day-old leaf explant of *V. erianthum* species that was inoculated with the A13 strain. The transformed hairy root colons were confirmed by PCR using *rol*B gene-specific primers. To optimize hairy root growth and avoid tissue browning, hairy roots were cultured in various media containing different antioxidants and improver agents (including ascorbic acid, citric acid, and NAA). The results showed that the highest fresh growth index (20.42) and the lowest tissue browning (9%) as well as total phenol (8.51 mg GA/g DW), and total flavonoid content (4.42 mg QUE/g DW) were obtained in medium B5 with 1.5 mg/l NAA.

## Introduction

The genus *Verbascum* (Mullein) belongs to the family Scrophulariaceae, principally a biennial herb with a deep taproot, rosette leaves in the first year, and spikes of flowers in the second year^1^. With 42 species, Iran is a large biodiversity center of the genus *Verbascum*. Twenty species of them are found in the West and East Azerbaijan provinces. *V. stachydiforme* is described as an endemic species and, *V. erianthum* is defined as a local species belonging to the Irano-Turanian region^2^.

The different organs (flower, leaf, and root) of mullein have long been used as anti-inflammatory agents in the treatment of hemorrhoids and respiratory diseases because they contain biologically active molecules such as the iridoid (aucubin and catalpol) and phenylethanoid glycosides (forsythoside B, verbascoside and leucosceptoside B), which are the main and important bioactive molecules found in *Verbascum* spp^3,4^. Verbascoside (acetoside) has been found in over 200 species, but it has been found primarily in *Verbascum* species and is known for its pharmacological effects, including anti-inflammatory, antiviral, antioxidant, antinephrotic, hepatoprotective, and neuroprotective effects^5^. This compound is a promising agent for skin disorders such as UV-induced non-melanoma cancers. However, the accumulation of small amounts of verbascoside in plants has limited the use of this compound for disease treatment. Therefore, it is essential to employ strategies that result in increased production and accumulation of this valuable medicinal compound. Hairy root culture system is an effective method to increase the production of this valuable phytochemical compound^6^. Acetoside is produced by in vitro plant culture systems, including hairy root cultures (HRCs)^5,7^, In the study conducted on *the V. xanthophoeniceum*, it was found that the amount of verbascoside produced in the hairy roots was six times higher than the intact plant^8^.

Sonication-assisted *Agrobacterium*-mediated transformation (SAAT) has been employed in hairy root induction of *V. xanthophoeniceum*^8^, *V. nigrum*^9^ and *V. eriophorum*^10^. The first step to use the benefits of hairy root culture is to optimize the induction of hairy roots in the studied plant. Obtaining an effective method for the production of hairy roots in this plant can be used in future studies to investigate the biosynthetic pathway, metabolite engineering and gene transferring, and metabolite farming of this valuable plant.

The transgenic performance of *A. rhizogenes* is influenced by the age of the explant, the bacterial strain, the timing of co-culture, the different plant species, the method of infection, and the composition of the culture medium^11^. Increasing growth and secondary metabolite production requires optimizing the composition of the hairy roots culture medium. It seems that changing the amount and the type of nutrients in the culture medium has a significant impact on the growth of hairy root lines^10,11^.

Despite the considerable species diversity of the *Verbascum* genus in Iran until now there has been no study on their hairy root induction. The present investigation was conducted to optimize conditions for the initiation and establishment of HRCs in two species of *Verbascum*.

## Results and discussion

**Figure 1:**
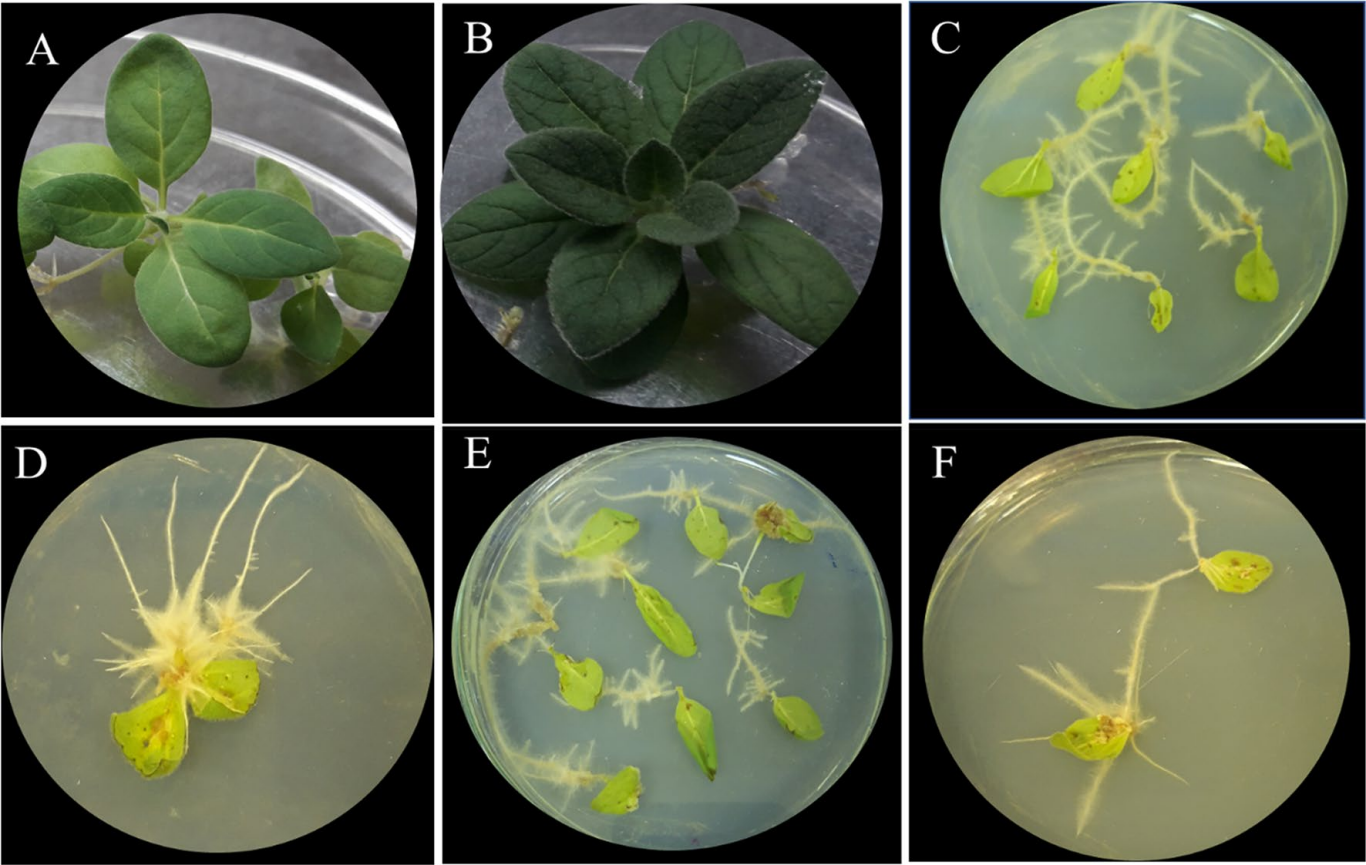
*Verbascum* species: (**A**): *V. erianthum*. (**B**): *V. stachydiform* mother plants*.* Hairy root induction in transformed explants (**C**): by ATCC15834 strain, (**D**): by A4 strain, (**E**): by A7 strain, (**F**): by A13 strain.

According to the best of our knowledge, until now, there have been no such reports to induce hairy roots in *Verbascum* species belonging to Iran (endemic species). So there was no efficient protocol for the induction of hairy roots and their establishment in these species. By examining different methods and using the SAAT method described by Georgiev et al.^8^, we developed an efficient protocol (direct infection with 3 wounds) for *V. erianthum* and *V. stacydiforme* (Fig. 1 A and B) hairy root culture induction.

### PCR analysis of transgenic hairy roots

**Figure 2:**
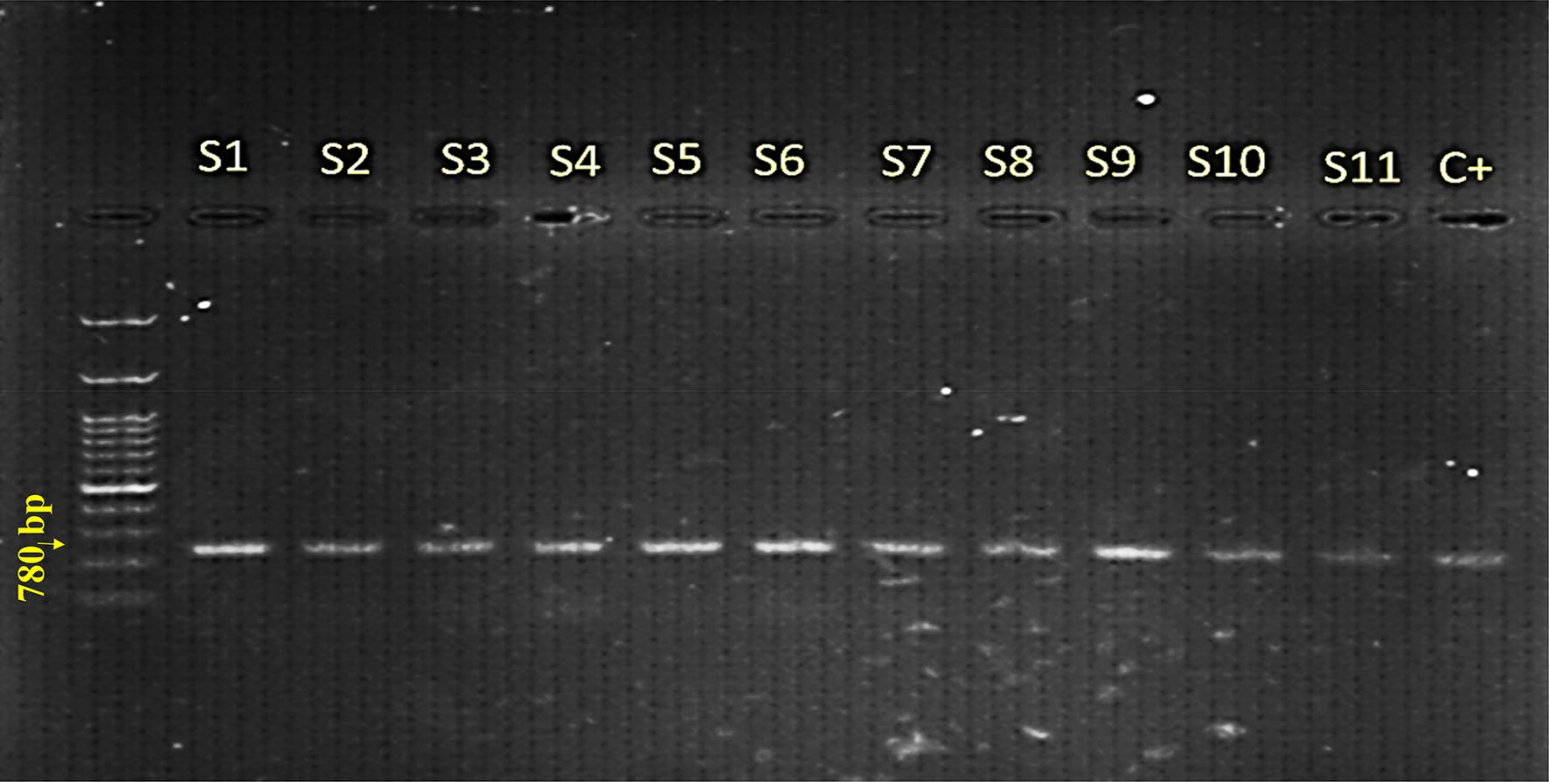
PCR amplified DNA fragments in size (780 bp) using specific primers for the rolB gene of *A. rhizogenes* on *Verbascum* hairy root DNA. M: 100 bp DNA Ladder, S1- S11: hairy root lines, C^+^: *A. rhizogenes* plasmid as a positive control.

The results of the polymerase chain reaction showed the presence of the expected band of 780 bp related to the *rol*B gene in the transformed roots in different explants used *by Agrobacterium rhizogenes* strains, which confirms the transfer T-DNA region of the bacteria plasmid to the hairy root genome. DNA templates from untransformed roots (used as controls) did not show any amplification (Fig. 2, Fig. 1S and Fig. 2S).

### Optimization of conditions for *A. rhizogenes* transformation

#### Effect of infection method, co-cultivation time, and explant type on transformation efficiency

**Figure 3:**
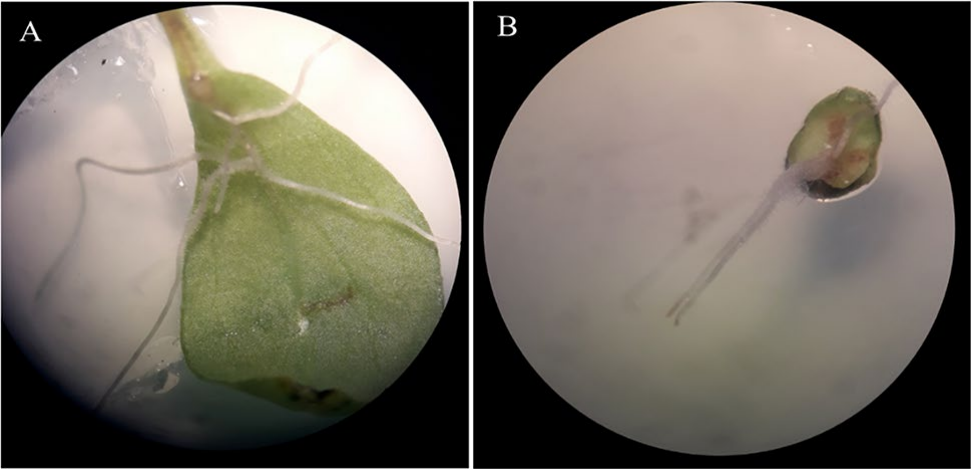
Hairy root induction by *A. rhizogenes* (ATCC15834 strain) in *V. erianthum* in leaf explant. (**A**) and hypocotyl explant (**B**) after 16 days with syringe needle infection method.

In this study, hairy roots were observed 16 to 18 days after transformation (Fig. 3). The results showed a significant difference in transformation efficiency among the treatments (*P* < 0.01) (Table1). The transformation efficiency ranged from 0 to 68.18, with the highest being in the leaf sample that had direct infection with 3 wounds and 72 h of co-cultivation time. The transformation efficiency in leaf samples with direct infection and 3 wounds at 48 h was 62.12. Leaf samples treated with SAAT for 45 s at 48 and 72 h of co-cultivation time showed transformation efficiencies of 48.48 and 51.09, respectively (Table 1). During the flooding method, the induction of hairy roots had the lowest success rate in both leaf and hypocotyl explants. Additionally, their growth was slow and eventually resulted in browning and death. Previous studies have demonstrated that efficient genetic transformation of the *Verbascum* genus with *A. rhizogenes* can be achieved using the SAAT protocol (consisting of 45-s sonication treatments) as described by Gergive et al. in^8^. Marchev et al. in^10^ used both the direct infection and SAAT methods and reported that they were able to induce hairy roots with a 50% infection frequency. However, only the colonies generated by the direct infection method were capable of growing. Throughout our study, we discovered that the effectiveness of genetic transformation through syringe needle infection hinges on the number and location of wounds made on the explant. Specifically, explants with five wounds experienced a significant decrease in hairy root induction, and most of them died. By increasing the number of wounds on the explant, the induction of hairy roots can be reduced. However, this method can trigger a plant defense response called the hypersensitive reaction (HR), which causes tissue necrosis, cell death, and accumulation of antimicrobial agents. Moreover, the transfer of T-DNA can lead to necrosis and cell death, ultimately decreasing the efficiency of transgenic cell regeneration and resulting in fewer clones with transgenic cells^12–14^. In order to achieve successful transformation, the petiole should be the site of the wound, and the veins and leaf blades should remain undamaged and untouched by the syringe. This is likely due to the abundance of vessels containing water and nutrients in the petiole, which are necessary for the production and growth of hairy roots^15,16^. As mentioned, the highest percentage of hairy root induction was observed within 72 h of the co-culture period (Table 1**)**, as reported by other studies^11,17^. The process of genetic transformation using *A. rhizogenes* involves the transfer of the T-DNA region from the plasmid of the bacteria into the nucleus of the plant, where it becomes integrated into the host plant's genome. Therefore, the length of the co-culture period is a crucial factor in determining the success of the transformation^11,18^.

**Table 1:** ANOVA analysis and means comparison of the effect of explant type, infection method, and co-cultivation time on Transformation efficiency.

Explant type	Infection method	Co-culture periods (h)	Transformation efficiency
Leaf	Direct infection 3 wounds	48	62.12 ± 1.51^b^
		72	68.18 ± 2.62^a^
	Direct infection 5 wounds	48	4.51 ± 2.62^f–h^
		72	7.57 ± 3.03f^g^
Hypocotyl	Direct infection 3 wounds	48	37.50 ± 3.60^d^
		72	39.58 ± 1.04^d^
	Direct infection 5 wounds	48	6.25 ± 1.80^f–h^
		72	5.20 ± 1.04^f–h^
Leaf	Flooding 5 min	48	1.42 ± 1.51^gh^
		72	4.54 ± 2.64^f–h^
	Flooding 10 min	48	0.00 ± 0^h^
		72	1.51 ± 1.02^gh^
Hypocotyl	Flooding 5 min	48	3.25 ± 1.80^f–h^
		72	4.1 ± 2.08^f–h^
	Flooding 10 min	48	2.01 ± 1.04^gh^
		72	7.29 ± 1.04^f–h^
Leaf	SAAT 45 s	48	48.48 ± 4.00^c^
		72	51.09 ± 2.62^c^
	SAAT 80 s	48	24.24 ± 3.03^e^
		72	27.01 ± 2.62^e^
Hypocotyl	SAAT 45 s	48	2.08 ± 2.08^gh^
		72	4.01 ± 2.08^f-h^
	SAAT 80 s	48	10.41 ± 1.04^f^
		72	8.33 ± 2.08^fg^
ANOVA df (Treatments 23; Error: 48; Total 71)			**

The similar letters are not statistically significant.

**Significant at *p* < 0.01.

#### Effect of explant age, different species, bacterial strain, and IBA on transformation efficiency

After determining the most effective infection method (direct injection with three site wounds), the ideal explant (leaf), and the co-cultivation time (72 h), the second section of the first experiment was planned and carried out. Leaf explants with different ages (14, 21, and 28 days) of *Verbascum* species were incubated with different strains of *A. rhizogenes* to find the best combination treatment for transformation efficiency (Fig. 1 C–F).

The findings indicate a notable variation in transformation efficiency (TE), average number of hairy roots (ANH), and secondary branche number (SBN), between the various treatments (P < 0.01). ANOVA of individual and combined effects of species type, explant age, and bacterial strain on TE, ANH, and SBN was statistically significant (Table 2). As shown in Table 3 the highest hairy root TE (80.55, 70.83%) was observed in the treatment combination of *V. erianthum* species, A13 and A7 strains, and 21-old day leaf explant. The TE of hairy roots varied among the treatments, ranging from 0 to 80.55%. For *V. erianthum*, the range was between 1.38–80.55%. The transfer of T-DNA from *A. rhizogenes* to the host plant depends on variable factors such as genotype, explant age, bacterial strain, and signaling molecules^19,20^. Among *V. erianthum* and *V. stachydiform* species the highest TE, the highest ANH per explant, and highest the SBN of the hairy root (N/cm) were detected in *V. erianthum* species. Each plant species has a different cell wall structure, physiological state, and molecular markers, which may lead to differences in the ability of species to be transformed^21,22^. The process of delivering T-DNA into plant cells genome requires the cooperation of bacteria and cells, and the identification of suitable plant cells by bacteria occurs through the mechanism of chemotaxis. In the next step, the type of receptors on the plant cell wall is important for bacterial entry into the plant cell^23^. Among the evaluated strains and explant ages, the A13 and A7 strains were suitable for providing high levels of transformation rates in all explant ages. This study found that various *Agrobacterium* strains had different abilities in gene transfer to plant cells^19,24,25^. A13 is a wild-type strain of *Agrobacterium* and belongs to the mikimopine type. Its Ri plasmid contains 12 repeats of T-DNA transfer stimulator sequences (TSS), and given the role of the TSS sequence in T-DNA transfer, this strain is expected to have high transgenic performance^20,26,27^. The morphology and growth rate of the hairy roots obtained from different strains of *A. rhizogenes* were significantly different. The highest ANH per explant (9 ± 0.94) and the highest SBN of hairy root per centimeter (15.00 ± 1.97) were observed in a 21-day-old explant of *V. erianthum* incubated with strain A4. According to previous studies, the A4 strain of *A. rhizogenes* has been found to be highly effective in promoting transformation in dicotyledonous plants^21^. This difference in transformation and morphology is related to the plasmids integrated into the bacterial strain, as well as the differential expression of T-DNA genes of the transgenic roots, the number of inserted T-DNA segments, the number of multiple copies of inserted T-DNA, and the position of the T-DNA in the plant genome^23,27^.

**Table 2:** ANOVA analysis of the effect of species, explant age, bacterial strain, and IBA hormone on transformation efficiency (TE), average number of hairy roots (ANH), and secondary branches number (SBN).

Source of variation	df	TE	ANH	SBN
Species	1	**	**	**
Explant age	2	**	**	**
Bacterial strain	3	**	**	**
IBA hormone	1	ns	ns	ns
Block	2	**	**	ns
Species * explant age	2	**	**	**
Species * bacterial strain	3	**	**	**
Species * IBA hormone	1	ns	ns	ns
Explant age * bacterial strain	6	**	**	**
Explant age * IBA hormone	2	ns	ns	ns
Bacterial strain * IBA hormone	3	ns	ns	ns
Species * explant age * bacterial strain	6	**	**	**
Explant age * bacterial strain * IBA hormone	6	ns	ns	ns
Species * bacterial strain * IBA hormone	3	ns	ns	ns
Species * explant age * IBA hormone	2	ns	ns	ns
Species * explant age * bacterial strain * IBA hormone	6	ns	ns	ns
Error	94			
Total	143			

*Ns* not significant.

**Significant at *p* < 0.01.

**Table 3:** Effect of species, explant age, and bacterial strain on transformation efficiency (TE), average number of hairy roots (ANH), and secondary branches number (SBN).

		Transformation efficiency		Average number of hairy roots per explant		Secondary branches number (N/cm)	
	Species	*V. erianthum*	*V. stachydiforme*	*V. erianthum*	*V. stachydiforme*	*V. erianthum*	*V. stachydiforme*
Explant age	Bacterial strain						
14 day	ATCC15834	44.44 ± 1.70^de^	19.44 ± 2.77^g–j^	1.66 ± 0.47^e–h^	2.00 ± 0.47^ef^	6.00 ± 1.22^e-g^	4.16 ± 0.54^f−g^
	A13	58.33 ± 2.15^bc^	6.94 ± 2.565^i–l^	4.33 ± 0.5^d^	1.50 ± 0.5^e–h^	10.16 ± 2.1^cd^	8.00 ± 1.54^c–e^
	A4	33.33 ± 2.17^ef^	9.71 ± 2.56^i–l^	7.33 ± 0.81^b^	1.83 ± 0.02^e–g^	8.00 ± 1.31^c-e^	3.50 ± 0.25^g−k^
	A7	48.61 ± 2.56^cd^	12.49 ± 2.84^ h–l^	1.66 ± 0.5^e–h^	1.16 ± 0.25^f–j^	4.83 ± 0.45^e-i^	2.16 ± 0.00^h–l^
21 day	ATCC15834	66.66 ± 3.04^b^	19.44 ± 2.84^ g–j^	2.5 ± 0.0^e^	1.83 ± 0.0^e–g^	10.83 ± 1.01^bc^	5.50 ± 0.75^e–h^
	A13	80.55 ± 2.77^a^	29.19 ± 2.77^ fg^	5.66 ± 1.2^c^	2.00 ± 0.00^ef^	14.16 ± 2.87^ab^	7.66 ± 1.31^c–e^
	A4	52.77 ± 2.77^ cd^	4.1 ± 1.75^kl^	9 ± 0.94^a^	0.50 ± 0.00^h–j^	15.00 ± 1.97^a^	3.33 ± 0.21^g−l^
	A7	70.83 ± 3.56^ab^	19.44 ± 3.14^ g–j^	1.33 ± 0.41^e–i^	1.33 ± 0.5^e–i^	7.00 ± 0.94^d–f^	5.00 ± 0.91^e–i^
28 day	ATCC15834	22.22 ± 4.12^f–i^	5.53 ± 0.34^kl^	1 ± 0.0^f–j^	0.66 ± 0.00^g–j^	1.66 ± 0.41^i–l^	1.00 ± 0.0^i–l^
	A13	15.28 ± 1.38^h–k^	0.0 ± 0^ l^	1.16 ± 0.0^f–j^	0.00 ± 0.00^j^	2.16 ± 0.46^h–l^	0.00 ± 0.0^l^
	A4	1.38 ± 0^l^	15.28 ± 2.14^ h–k^	0.16 ± 0.0^ij^	1.83 ± 0.42^e-g^	0.16 ± 0.0^kl^	1.16 ± 0.21^i−l^
	A7	25 ± 2.15^f–h^	16.66 ± 1.87^g–k^	1.16 ± 0.47^f–j^	1.00 ± 0.0^f–j^	1.83 ± 0.0^i–l^	2.5 ± 0.00^ h−l^

In each column, the similar letters are not statistically significant.

**Figure 4:**
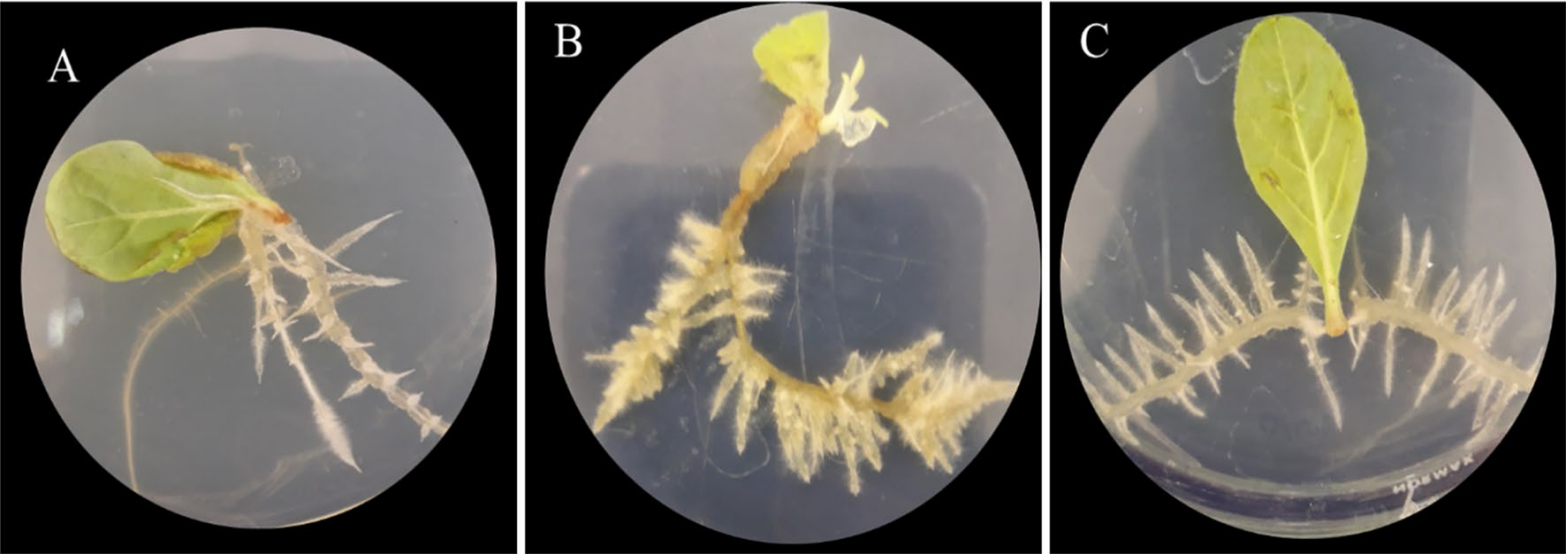
Hairy root morpho-types in MS media supplemented with IBA hormone: (**A**): Thick roots with few branches, (**B**): Callous + hairy roots, (**C**): Callusing roots.

As a result, the TE changed with the age of the explant, when the 14 and 28-day-old leaf explants were used, the transformation rates decreased significantly, and the 21-day-old leaf explants were found to be better for transformation efficiency in *Verbascum* species (Table 1). The response to transformation was significantly influenced by the age of the explant. Tissues with younger cells have smaller vacuoles, therefore the probability of TE increases. The addition of 1 mg/l IBA hormone to MS media did not result in a significant increase in TE, ANH, SHN in centimeters, according to the study. It seems that the hormone IBA plays a significant role in undesirable morphologies, such as thick roots with few branches. Also, callous-like roots were observed in MS media supplemented with IBA (Fig. 4). Several studies have reported the existence of such morphologies in hairy roots^28,29^.

This difference in hairy root growth and morphology is caused by different bacterial strains with various *rol* genes actions. It seems that the expression of these genes alters the internal level of auxin and cytokinin hormones or the sensitivity of plant cells to respond to these hormones. The plasmid of A4 (which belongs to the agropin strains) contains both TR and TL regions. The *aux* genes of TR-DNA could lead to additional auxin production, so internal auxin has a special effect on hairy root growth, lush branching, and stopping linear growth^16,30,31^. In this study, the growth variability among hairy root lines was visible, so that lines with very rapid growth and luxuriant branching morphology could be selected (data not shown).

The A13 strain produced higher-quality hairy roots compared to the A4 strain. The growth of A4-induced hairy roots was not optimal without the addition of auxin to the culture medium. Both strains contain two types of T-DNA regions (TR-DNA and TL-TDNA) on the Ri plasmid, which are transferred independently into the plant cell^32^. If the TR-DNA, containing *aux* genes, is not inserted correctly into the plant genome or its expression is unsuitable based on the insertion site or number of copies, the hairy roots may become auxin-auxotrophic^33^. For the second experiment, the L2.2 hairy root colonies that had the best morphology, abundant branching, lower doubling time, and expansion on the MS medium were selected and incubated with the A13 strain.

#### Effect of different organic compounds and culture media on hairy roots establishment

**Figure 5:**
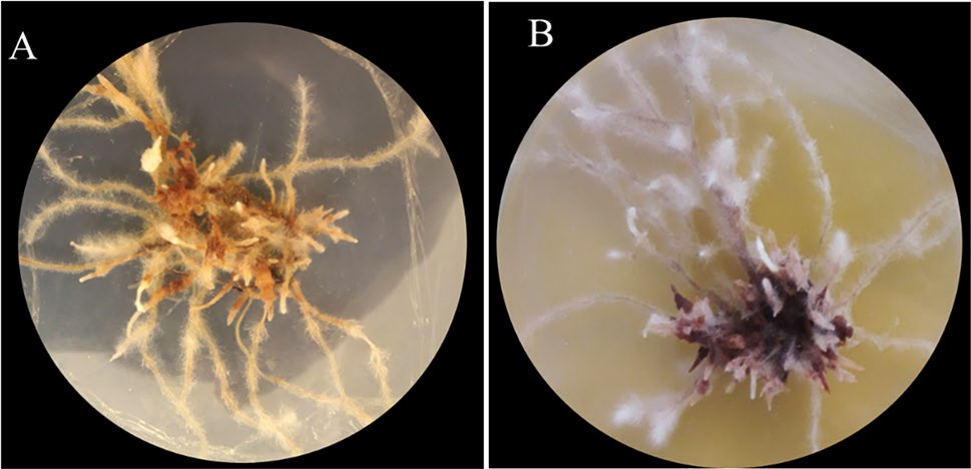
The status of browning of the samples of hairy roots (**A**): Hairy roots become necrotic and brown after subculture. (**B**): Prevention of hairy root browning in B5 culture medium supplemented by 1.5 mg/kg NAA.

During our preliminary studies, we discovered that *Verbascum* hairy roots experienced necrosis and browning after being subcultured to a new medium (Fig. 5A), which hindered their stability. Therefore, to obtain a high biomass of hairy roots, it was necessary to first address this issue by optimizing and providing a standard culture medium for them. This browning occurs due to the hairy roots' rich content of various phenolic compounds, which are quickly oxidized by the enzyme polyphenol oxidase (PAL), leading to tissue necrosis. This inhibits the accumulation of hairy root biomass^34^*.* The activity of PAL increases under stressful conditions, such as mechanical damage. It is worth noting that before our study, there were no references to the browning and necrosis of hairy roots in the *Verbascum* genus. This could be attributed to the genetic diversity in nature.

Our study found that different media compositions had a significant effect on hairy root browning, fresh weight, growth index, and phenolic compounds based on ANOVA analysis (Table 4).

**Table 4:** ANOVA analysis and means comparison of the effect of organic compounds, NAA, and culture media on hairy root establishment.

	concentration	culture medium	browning (%)	FGI	TPC	TFC
Ascorbic acid	100 mg/kg	MS	76.0 ± 0.57^ab^	6.07 ± 0.04^kl^	20.29 ± 0.18^d^	9.64 ± 10^c^
		½ MS	62.33 ± 0.88^c^	6.28 ± 0.10^kl^	17.54 ± 0.24^f^	7.27 ± 0.9^f^
		B5	51.66 ± 1.02^ef^	8.51 ± 0.20^ fg^	14.25 ± 0.18^ h^	7.47 ± 0.12^ef^
	150 mg/kg	MS	71.33 ± 1.4^b^	5.6 ± 0.26^ l^	21.49 ± 0.18^c^	8.45 ± 0.33^d^
		½ MS	55.33 ± 1.76^de^	7.39 ± 0.24^ h-i^	15.66 ± 0.17^g^	8.15 ± 0.19^de^
		B5	50.33 ± 0.88^ef^	7.92 ± 0.38^ g-i^	13.55 ± 0.33^h^	7.34 ± 0.15^ef^
Citric acid	50 mg/kg	MS	79.00 ± 1.52^a^	6.54 ± 0.32^i-l^	23.99 ± 0.13^a^	11.38 ± 0.15^a^
		½ MS	60.66 ± 0.33^cd^	8.45 ± 0.13^f–h^	23.39 ± 0.22^ab^	10.51 ± 0.22^b^
		B5	46.66 ± 0.88^fg^	11.48 ± 0.14^e^	17.66 ± 0.11^f^	9.60 ± 0.09^c^
	75 mg/kg	MS	76.33 ± 0.33^ab^	7.08 ± 0.26^i–k^	22.48 ± 0.15^bc^	11.53 ± 0.07^a^
		½ MS	62.0 ± 0.34^c^	9.18 ± 0.20^f^	19.24 ± 0.33^de^	10.33 ± 0.26^bc^
		B5	38.66 ± 0.88^hi^	13.53 ± 0.19^d^	18.60 ± 0.13^ef^	8.41 ± 0.18^d^
NAA	1 mg/l	MS	44.33 ± 0.66^gh^	16.28 ± 0.33^c^	10.94 ± 0.05^i^	6.7 ± 0.13^fg^
		½ MS	31.66 ± 0.89^i^	16.49 ± 0.07^c^	11.57 ± 0.23^i^	6.2 ± 0.12^g^
		B5	16.33 ± 1.45^l^	19.36 ± 0.23^ab^	9.39 ± 0.14^j^	5.20 ± 0.12^hi^
	1.5 mg/l	MS	16.33 ± 1.45^ij^	17.34 ± 0.16^c^	10.64 ± 0.23i	4.84 ± 0.08^hi^
		½ MS	25.0 ± 1.52^k^	19.31 ± 0.32^b^	10.69 ± 0.33^i^	5.30 ± 0.14^ h^
		B5	9.0 ± 0.57^m^	20.42 ± 0.16^a^	8.51 ± 0.03^j^	4.42 ± 0.19^i^
ANOVA df (Treatments 17; Error: 36; Total 53)			**	**	**	**

In each column, the similar letters are not statistically significant.

**Significant at *p* < 0.01.

**Figure 6:**
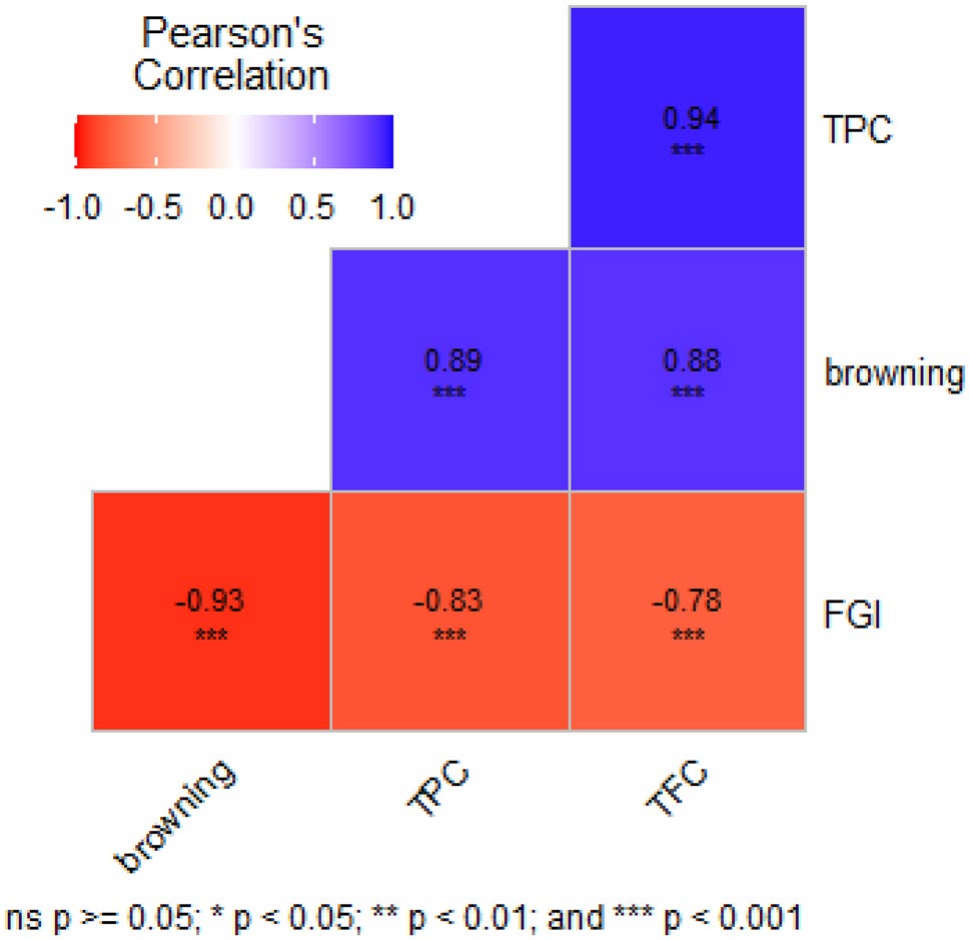
The correlation coefficient between hairy root browning, FGI, TPC, and TFC. positive correlation is displayed in blue and negative correlation in red.

Table 4 represents a summary of the effects of different media compositions, namely MS, 1/2MS, and B5, supplemented with different concentrations of antioxidant compounds and NAA, on hairy root browning, fresh weight, growth index, and phenolic compounds. The maximum percentage of hairy root browning (79%), total phenolic content (23.99 mg GA/g DW), and total flavonoid content (11.38 mg QUE/g DW) were observed in MS medium supplemented with 50 mg/kg citric acid. On the other hand, B5 medium supplemented with 1.5 mg/l NAA resulted in the lowest percentage of browning (9%) and the highest fresh growth index (20.42) of hairy roots. The use of a hormonal growth regulator (1.5 mg/kg NAA) in this study significantly prevented hairy root browning, allowing them to grow successfully (Fig. 5 A and B). According to Fig. 6, a significant negative correlation (*p*-value < 0.001) was observed between the growth index and the hairy root browning (− 0.93), total phenol (0.83), and total flavonoid (− 0.78) content. Therefore, with the increase in the amount of phenolic compounds, the hairy root browning also increases, and the growth index decreases.

Observations showed that the hairy root browning decreased with the increase in the concentration of NAA (from 1 to 1.5 mg/l) in MS, 1.2 MS, and B5 culture media 2.75, 1.24, and 1 time, respectively.

Despite the use of antioxidants, such as ascorbic acid and citric acid, at the concentration used in this study, tissue browning could not completely be eliminated, however, the rate of browning was reduced. Research has shown that the combination of ascorbic acid and citric acid effectively prevents tissue browning in *Litchi chinensis* explants^35^. Similarly, a mixture of ascorbic acid, citric acid, and PVP was found to be successful in inhibiting browning and reducing necrosis in *A. mango* explants^34^. The use of NAA as a growth stimulant for hairy roots in combination with sugar and auxins has also been successful in other studies^36^. The positive and effective role of auxins in promoting hairy root growth in chicory was investigated by Fathi et al.^17^. The study also found that transgenic hairy roots had a higher capacity for hormone uptake than non-transgenic roots. Another study by Gangopadhyay et al.^37^ investigated the effects of growth regulators on hairy roots and found that naphthaleneacetic acid (NAA) had the most significant effect on growth and secondary metabolite production in Plumbago indica hairy roots compared to gibberellic acid (GA3), indole butyric acid (IBA) and indoleacetic acid (IAA).

Based on our observations, different culture media were classified as B5 > ½ MS > MS, with B5 producing the most biomass and reducing tissue necrosis and browning. Hairy root growth was significantly influenced by the type and amount of nutrients in the culture medium. It was found that the B5 medium produced more biomass and reduced tissue necrosis and browning compared to MS and ½ MS culture media, culture media with high salt (MS) are not suitable for the growth of *V. erianthum* hairy roots. Nitrogenous compounds are important components of the culture medium that affect the growth response. Researchers have shown that the type of nitrogen, as opposed to its concentration, has a greater effect on hairy root growth^38^. The nitrogen in MS and ½ MS culture media is supplied by ammonium nitrate (NH_4_NO_3_) while in B5 it is supplied by potassium nitrate (KNO_3_). Increasing the potassium nitrate content acts as the sole source of nitrate in B5 media. Studies have shown that the optimal ratio of NH_4_^+^ to NO_3_^-^ affects the hairy root growth in *Valeriana officinalis*^39^, *Artemisia annua*^40^, and *Datura candida*^41^. Some reports show that a high concentration of ammonium in the MS culture medium leads to a reduction and inhibition of growth because ammonium is used before nitrate and causes a decrease in the pH of the culture medium^42–44^. Increasing the concentration of ammonium in *Atropa belladonna* hairy root culture caused a decrease in hairy root growth, but had a strong influence on the biosynthesis and accumulation of alkaloids^45^.

In a culture medium, a high concentration of one of the components can lead to stressful conditions and the production of reactive oxygen species, as a result, it stimulates PAL enzyme activity and increases the biosynthesis of antioxidant compounds (such as phenolic compounds)^46^. In the B5 culture medium, the potassium present in potassium nitrate helps to build thicker cell walls and increase the level of electrolytes in the cells, which leads to increased plant resistance. Another important difference between different culture media is the concentration of thiamine, ranging from 0.1 (MS) to 10.0 (B5) mg /l, which prepares plants for biotic and abiotic stress and is required by all cells for growth.

## Conclusion

This is the first report on the development and optimization of hairy root transformation in Iranian *Verbascum* species. Hairy root transformation efficiency reached 80.55% with a 21-day-old explant (*V. erianthum*) by the A13 strain (syringe needle infection method-3 wounds) gave rapid root growth, the protocol investigated in this work provides a robust system for *Verbascum* genus hairy root culture. This study shows that necrosis, which is often observed in hairy root tissue, is associated with hypersensitive defense responses induced by PAL activity under conditions of mechanical damage when hairy roots are subcultured in new media culture. Hairy root browning was limited by adding 1.5 mg/kg NAA to the B5 medium. It is also necessary to evaluate and screen the effects of different buffers, a combination of antioxidants (e.g. a mixture of ascorbic acid and citric acid), PVP, and activated charcoal with different mechanisms in *Verbascum* species to prevent hairy root browning and increase biomass production. The discovery of the important role of infection method, explant type and age, bacterial strain, and plant species on hairy root induction and growth in vitro in culture is a very important manipulation in the production of hairy root biomass in large-scale industrial applications for the genus *Verbascum*.

## Methods

### Collection of plant materials

The seeds of *V. erianthum* were collected from Mahabad (N: 36°44′04", E: 45°36′56", Altitude: 1364 m), and *V. stachydiforme* were gathered from Sardasht (N: 36°11′15", E: 45°30′07", Altitude: 1228 m), West Azerbaijan province, Iran. The seed sampling was collected with permission from the university, comply with relevant institutional, national, and international guidelines and legislation. The sampling comply according with the IUCN Policy Statement on Research Involving Species at Risk of Extinction and the Convention on the Trade in Endangered Species of Wild Fauna and Flora. The seeds were then washed with sterile distilled water and then immersed in sodium hypochlorite 5% (V/V) for 10 min, followed by ethanol 70% (V/V) for 1 min. After washing three times with sterilized distilled water, they were cultured in MS hormone-free culture medium^47^ with 3% sucrose (W/V) and 0.7% agar (W/V) and kept at a temperature of 25 ± 1 °C under a photoperiod of 16/8 h light/darkness. Both species were identified by Dr Atefeh Ebrahimi, Central Herbarium of Tehran University, School of Biology, College of Science, University of Tehran, Tehran, Iran. The plants with voucher specimen (no 1506 and 1507) were labeled at the herbarium of the Department of Horticulture of Urmia University, Iran.

### *Agrobacterium* strains and culture conditions

Different strains of *Agrobacterium rhizogenes* (ATCC 15,834, A13, A4, and A7) were provided from the microbial bank of the National Institute of Genetic Engineering and Biotechnology of Tehran. A colony of each bacterial strain was grown in a liquid LB culture medium^48^ containing 50 mg/l of rifampicin antibiotic overnight in a shaker incubator (28 °C and 100 rpm) in dark to mid-log-phase (OD 600 nm) = 0.6–0.8. The bacterial suspensions were collected by centrifugation for 10 min and re-suspended in a liquid inoculation medium (half-strength MS medium containing 3% sucrose).

### Experimental design and work planning

**Figure 7:**
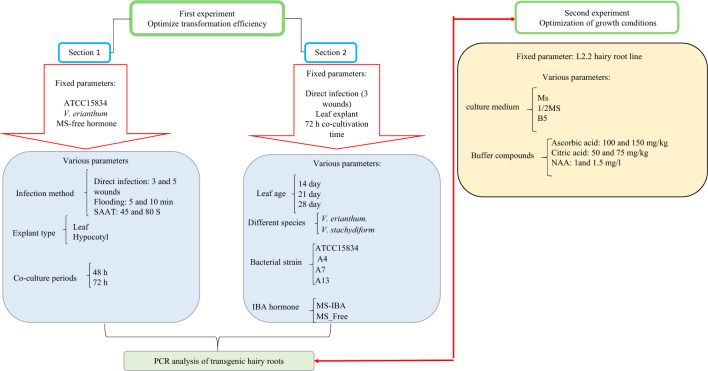
Workflow to optimize transformation efficiency and growth condition.

For the improvement of inducing and growing of hairy roots in *Verbascum* spp., two separate experiments were performed to optimize conditions for *A. rhizogenes* transformation and growth conditions. The steps of the experiment are shown in the diagram (Fig. 7).

### Optimization of conditions for *A. rhizogenes* transformation

The first experiment to improve *Agrobacterium*-mediated genetic transformation was performed in two separate parts:

I) Optimization of three factors, including (1) infection method (Direct injection with 3 wounds, direct injection with 5 wounds, flooding for 5 min., flooding for 10 min., Sonication-assisted *Agrobacterium*-mediated transformation (SAAT) for 45 s, SAAT for 80 s), (2) Co-culture periods (48 and 72 h), and (3) explant types (leaf and hypocotyl) were investigated with *A. rhizogenes* ATCC15834 in *V. erianthum.*

II) In the second experiment, optimization of four factors, including (1) Explant age (14, 21, and 28 days), (2) two species of *V. stachydiforme* and *V. erianthum* (3) four strains of *A. rihizigenes* (ATCC15834, A13, A4, and A7), and (4) two culture MS media (with or without IBA) were evaluated.

### Optimization of growth condition

The most rapidly growing hairy root line chosen from the previous stage was utilized to optimize the culture medium for increased biomass and to prevent tissue browning. The factors and their levels were as follows: (1) Improver and antioxidant compounds, including NAA (1 and 1.5 mg/l); ascorbic acid (100 and 150 mg/l); and citric acid (50 and 75 mg/l) and 2) were added to three basic culture media (MS, 1/2 MS, and B5).

### Details of genetic transformation with *A. rhizogenes*

Hypocotyl and leaf explants prepared from in vitro seedlings were used to induce hairy roots. In SAAT, ultrasonic treatments were performed using an Elmasonic E 120 Hz device (Elma Schmidbauer GmbH, Germany) at a temperature of 20 °C and an intensity of 120 Hz based on the procedure described by Georgiev et al.^8^.

After treating the infection as described above, the infected explants were placed on filter paper to remove any excess bacteria and then directly transferred to the MS solid medium. Co-cultivation was conducted under dark conditions at a temperature of 25 °C for 48 to 72 h. Following this, the explants were washed five times with sterile water before being transferred to the MS solid medium supplemented with 250 mg L^–1^ cefotaxime. After three weeks, the transformation efficiency was recorded. To ensure bacteria-free hairy roots, they were subcultured onto a hormone-free solid MS medium every three weeks, which contained cefotaxime antibiotics.

### PCR analysis of hairy roots

To identify the presence of Ri T-DNA, genomic DNA was isolated from transgenic hairy root lines using the CTAB method^49^. Negative control samples were taken from non-transformed adventitious roots, while positive control samples were taken from the *A. rhizogenes* plasmid (by sarikaya topal 2004 method). To detect the *rol* B gene of *A. rhizogenes* in the hairy roots, the following primer sets were used for polymerase chain reaction (PCR):

Forward -5'-ATGGATCCCAAATTGCTATTCCT TCCACGA-3'.

Reverse-5'-TTAGGCTTCTTTCTTCAGGTTTA CTGCAGC- 3'.

To run the PCR program, start with an initial denaturation step for 5 min. at 94 °C. Then, perform 35 thermal cycles consisting of denaturation at 94 °C for 1 min., annealing at 60 °C for 80 s, and extension at 72 °C for 90 s. Finally, complete the program with a step at 72 °C for 10 min. To examine the PCR amplicons, use electrophoresis on agarose gels with a concentration of 1.5% (w/v) and stain with ethidium bromide.

### Extraction and Determination of total phenolic and flavonoid content

Hairy roots were dried at room temperature, then 1.0 g of dried powder was mixed with 20 ml of methanol (80%) and extracted using an ultrasonic method (Elmasonic E 120 Hz, Elma Schmidbauer GmbH, Germany) at 30 °C for 30 min.

To determine the total flavonoid content in hairy root extracts, the aluminum chloride method was utilized as described by Chang et al.^50^. For determining the total phenolic content of the extracts, the Folin–Ciocalteu reagent was used with a little modification based on the method reported by Meda et al.^51^.

### Growth measurement

The growth of hairy roots was determined through fresh weight measurement. For this means, the hairy roots were placed between the folds of the blotting paper to remove any extra moisture. The fresh growth index (FGI) was used to express the growth. It was expressed as follows:

FGI = (Final fresh weight of biomass—Initial fresh weight of inoculum) / Initial fresh weight of inoculum.

### Statistical analysis

All experiments were conducted in triplicate. The first part of the transformation experiment and the experiment of optimization of growth condition (the second experiment) were performed in a completely randomized design (CRD). A factorial design based on a completely randomized design was used for the second part of the transformation experiment. All data were the mean ± standard deviation (SD). One-way ANOVA tests were carried out with a 95% confidence limit (P ≤ 0.05) by using the SPSS 15 (SPSS, Chicago, IL) software package. The correlation coefficients were calculated using R Studio 4.1.1 software.

### Ethical statement

Plant sampling complies with the IUCN Policy Statement on Research Involving Species at Risk of Extinction and the Convention on the Trade in Endangered Species of Wild Fauna and Flora.

### Supplementary Information

Supplementary Figures.

## Data Availability

The datasets generated during and/or analyzed during the current study are available from the corresponding author on reasonable request.
