# Supplementary material for: Optimization of induction and hairy root culture establishment in two mullein species, Verbascum erianthum and Verbascum stachydiforme
Source: Sci Rep. 2024 Mar 7;14:5636. doi: 10.1038/s41598-024-56331-8 (PMC10920640; doi:10.1038/s41598-024-56331-8)
Supplement: Supplementary file 1 — Supplementary Figures. [file 41598_2024_56331_MOESM1_ESM.pptx]

## Slide 1
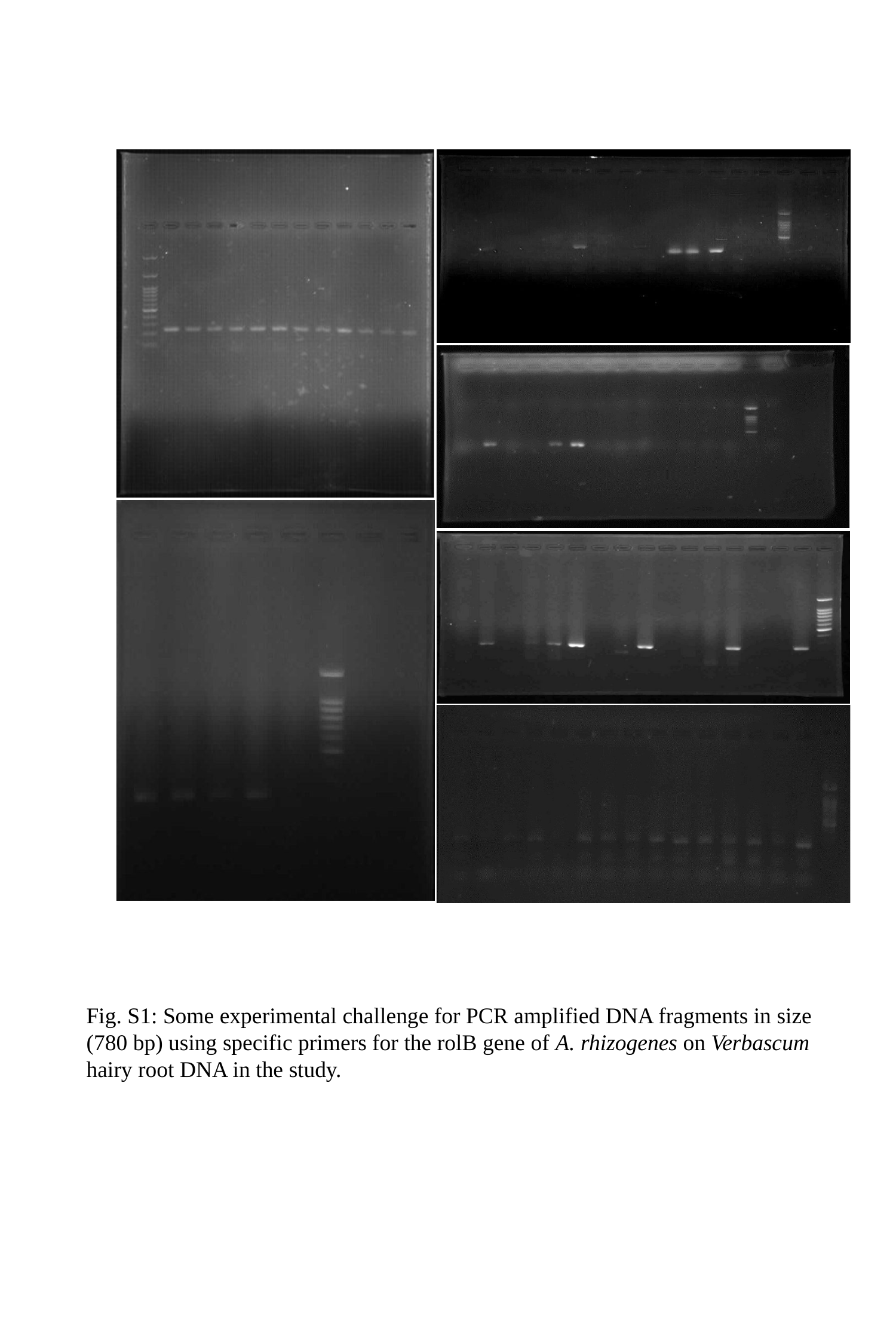

Fig. S1: Some experimental challenge for PCR amplified DNA fragments in size (780 bp) using specific primers for the rolB gene of A. rhizogenes on Verbascum hairy root DNA in the study.

## Slide 2
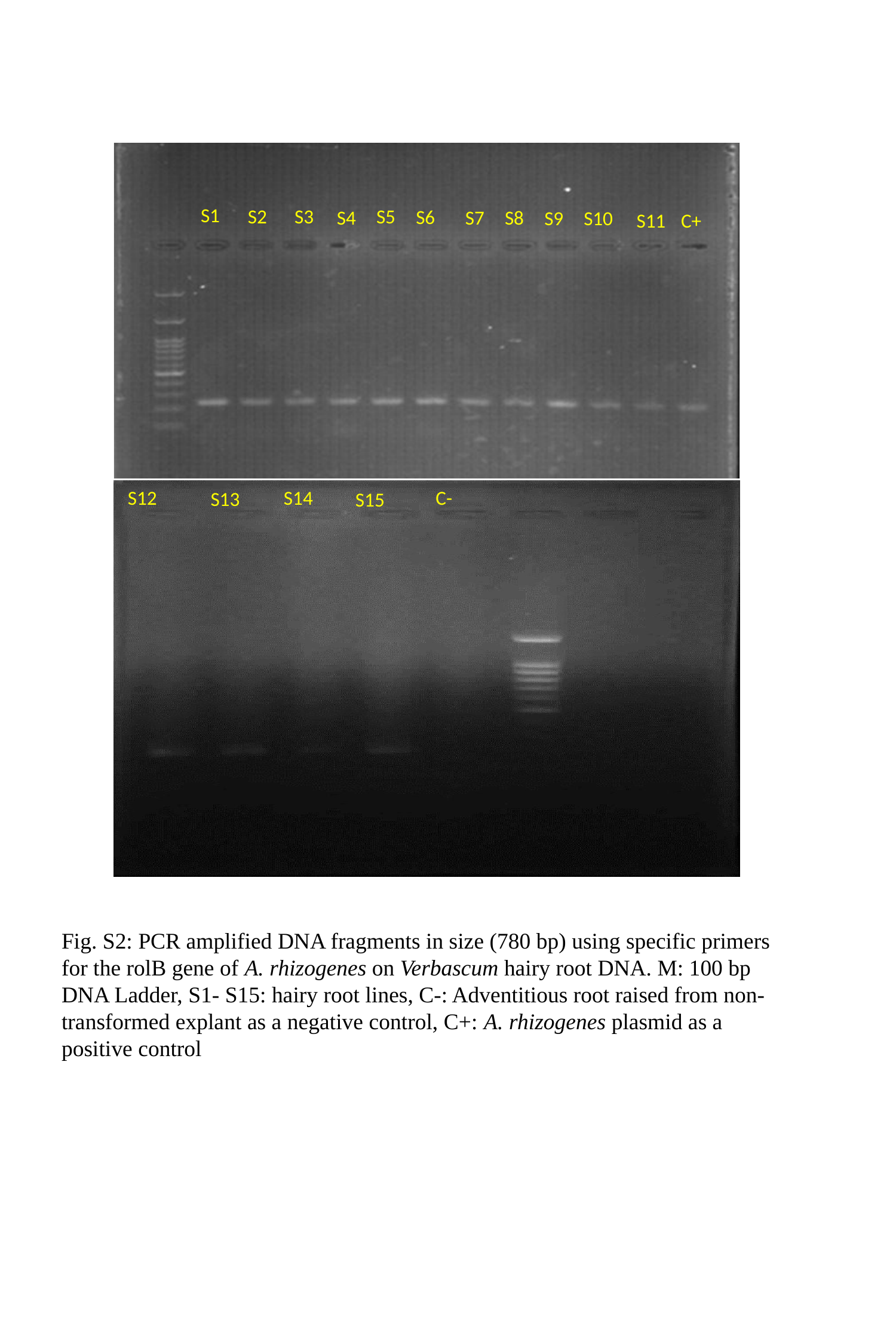

S1
S2
S3
S5
S6
S4
S7
S8
S9
S10
S11
C+
S12
S14
C-
S13
S15
Fig. S2: PCR amplified DNA fragments in size (780 bp) using specific primers for the rolB gene of A. rhizogenes on Verbascum hairy root DNA. M: 100 bp DNA Ladder, S1- S15: hairy root lines, C-: Adventitious root raised from non-transformed explant as a negative control, C+: A. rhizogenes plasmid as a positive control
